# Granulomatosis With Polyangiitis Unmasked by COVID-19 Infection: A Case Report

**DOI:** 10.7759/cureus.106729

**Published:** 2026-04-09

**Authors:** Adriana M Gomez, Carlos J Palencia

**Affiliations:** 1 Internal Medicine, Spartanburg Medical Center, Spartanburg, USA; 2 Internal Medicine, Nephrology, Endocrinology, Rheumatology, University of Carabobo, Valencia, VEN

**Keywords:** autoimmunity and covid, granulomatosis with polyangiitis (gpa), hemoptysis in covid-19, sars-cov-2 and covid-19, vasculitis

## Abstract

Granulomatosis with polyangiitis (GPA) is a type of anti-neutrophil cytoplasmic antibody (ANCA)-associated vasculitis (AAV), in which small- to medium-sized vessels are targeted. The incidence increases with age, and the most commonly affected systems include the sinonasal tract, lungs, and kidneys. Etiology remains unclear, although exposure to environmental triggers, including infections, has been hypothesized to initiate or unmask autoimmunity in genetically predisposed individuals. The nasal microbiome is also hypothesized to play a role in the disease process, although research is still ongoing.

In this report, we present a case of a 65-year-old female with a long history of nonspecific musculoskeletal, sinus, and gastrointestinal complaints, who developed multifocal pneumonia with hemoptysis and progressive pulmonary disease shortly after COVID-19 infection.

## Introduction

Recent evidence has highlighted the potential for viral infections to act as triggers of autoimmunity, particularly in genetically predisposed individuals. The global spread of COVID-19, caused by severe acute respiratory syndrome coronavirus 2 (SARS-CoV-2), has provided further insight into this phenomenon. Beyond its well-established respiratory manifestations, COVID-19 has been increasingly associated with immune dysregulation, characterized by excessive cytokine release, loss of self-tolerance, and the emergence of autoantibodies [1,2].

Among the autoimmune conditions reported in temporal association with COVID-19, vasculitides have gained particular attention. Granulomatosis with polyangiitis (GPA), a form of anti-neutrophil cytoplasmic antibody (ANCA)-associated vasculitis (AAV), is a rare but potentially life-threatening disease characterized by necrotizing granulomatous inflammation and small- to medium-vessel vasculitis, most commonly affecting the upper and lower respiratory tracts and kidneys. Although the exact etiology of GPA remains incompletely understood, environmental triggers, including infections, have long been implicated in disease onset and flares [3,4].

Emerging reports suggest that SARS-CoV-2 infection may precipitate or unmask autoimmune diseases such as GPA through several proposed mechanisms, including molecular mimicry, bystander activation, and persistent immune stimulation. These processes may promote the production of ANCAs and contribute to endothelial injury and systemic inflammation [5-7].

In this context, we present a case of newly diagnosed GPA following recent COVID-19 infection, aiming to contribute to the growing body of literature supporting a potential association between SARS-CoV-2 and the development of autoimmune vasculitis. This case underscores the importance of maintaining a high index of suspicion for autoimmune phenomena in patients presenting with atypical or persistent symptoms after COVID-19.

## Case presentation

A 65-year-old woman with a longstanding history of recurrent sinusitis and chronic abdominal pain presented to the Emergency Department with persistent respiratory symptoms and new-onset hemoptysis.

Over the preceding five to seven years, she had undergone extensive evaluation for her abdominal pain, including multiple contrast-enhanced computed tomography (CT) scans, laboratory studies, and endoscopic procedures (esophagogastroduodenoscopy and colonoscopy), all of which failed to identify a unifying diagnosis. The patient failed outpatient treatment with multiple courses of proton pump inhibitors, nonsteroidal anti-inflammatory drugs, and opioids. She also underwent several surgical interventions, including cholecystectomy, appendectomy, hernia repairs, and ultimately an exploratory laparotomy, none of which revealed a clear etiology or resolved her pain.

Approximately three to four weeks prior to presentation, the patient developed a mild productive cough associated with malaise, shortness of breath, fatigue, and chills. She tested positive for coronavirus disease 2019 (COVID-19) and was initially managed with supportive care, as she did not require supplemental oxygen and remained clinically stable. Due to persistent symptoms, she sought care at urgent care facilities on multiple occasions and was treated with oral antibiotics without improvement.

She subsequently presented to the Emergency Department with ongoing symptoms and new-onset hemoptysis. On initial evaluation, she was febrile, tachycardic, and intermittently hypoxic, and the remainder of her physical examination was unremarkable. Laboratory studies, as shown in Table 1, demonstrated leukocytosis, acute normocytic anemia, thrombocytosis, and elevated inflammatory markers. Procalcitonin levels were within normal limits.

**Table 1:** Results of admission tests WBC: white blood cells; Sed. rate: sedimentation rate; CRP: C-reactive protein

Test	Reference	Result
WBC	4.0-11.0 x 10^3^/uL	16.2 x 10^3^/uL
Hemoglobin	11.5-15.0 g/dL	10.5 g/dL
Platelets	135-400 x 10^3^/uL	497 x 10^3^/uL
CRP	0.0-0.6 mg/dL	19 mg/dL
Sed rate	0-30 mm/hrs	62 mm/hrs
Procalcitonin	0.02-0.25 ng/mL	0.10 ng/mL

Testing for influenza A and B, respiratory syncytial virus, and comprehensive pneumonia serologies was negative, as was methicillin-resistant and methicillin-sensitive *Staphylococcus aureus* screening.

**Figure 1:**
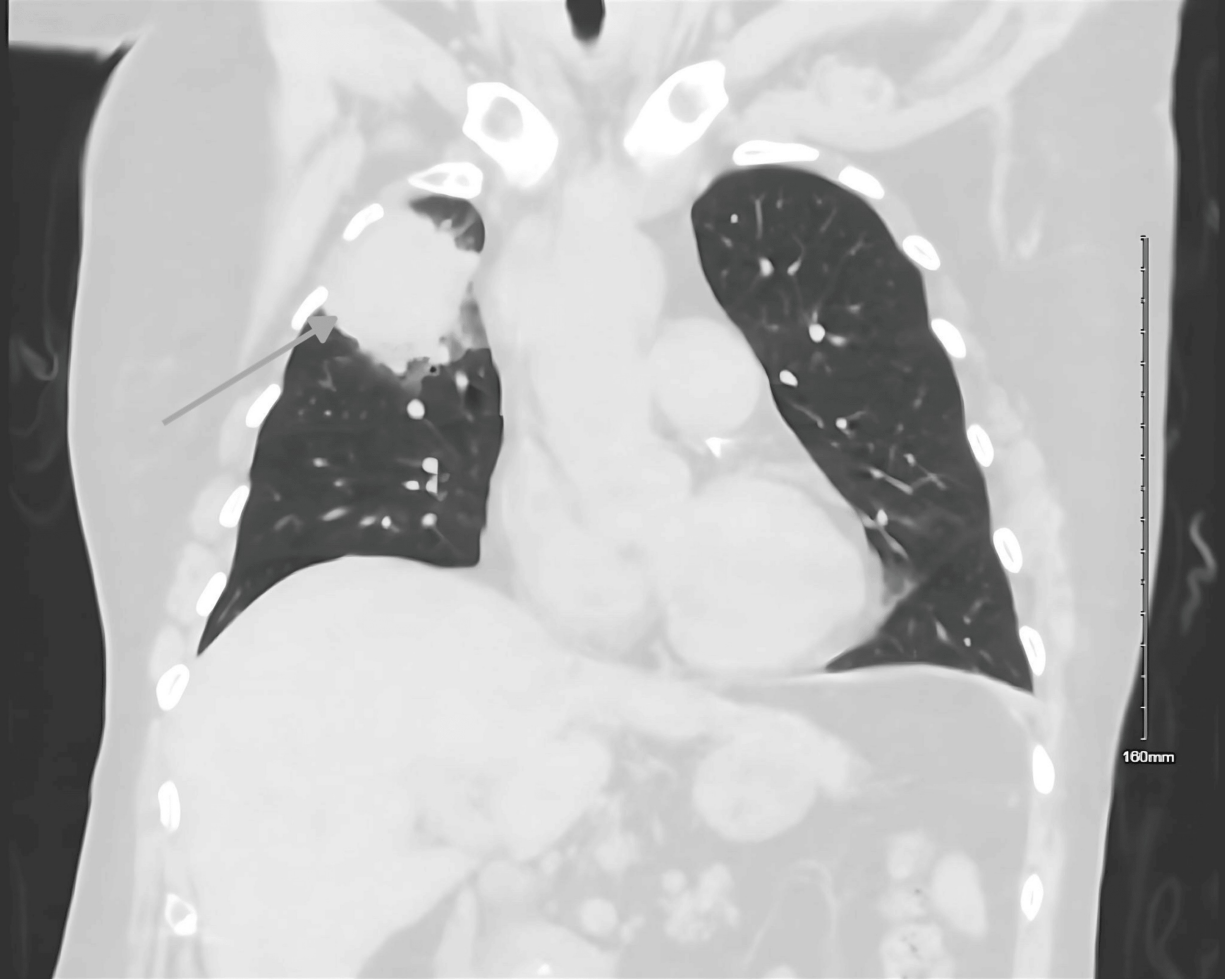
Computed tomography scan of the chest showing a large anterior right upper lobe masslike consolidation (gray arrow)

Contrast-enhanced CT of the chest revealed a large anterior right upper lobe mass-like consolidation with additional smaller bilateral pulmonary opacities, as seen in Figure 1. The differential diagnosis included multifocal pneumonia, malignancy, granulomatous disease, connective tissue disease, and vasculitis. Given her recent healthcare exposure, empiric broad-spectrum antibiotics were initiated for presumed healthcare-associated pneumonia.

**Figure 2:**
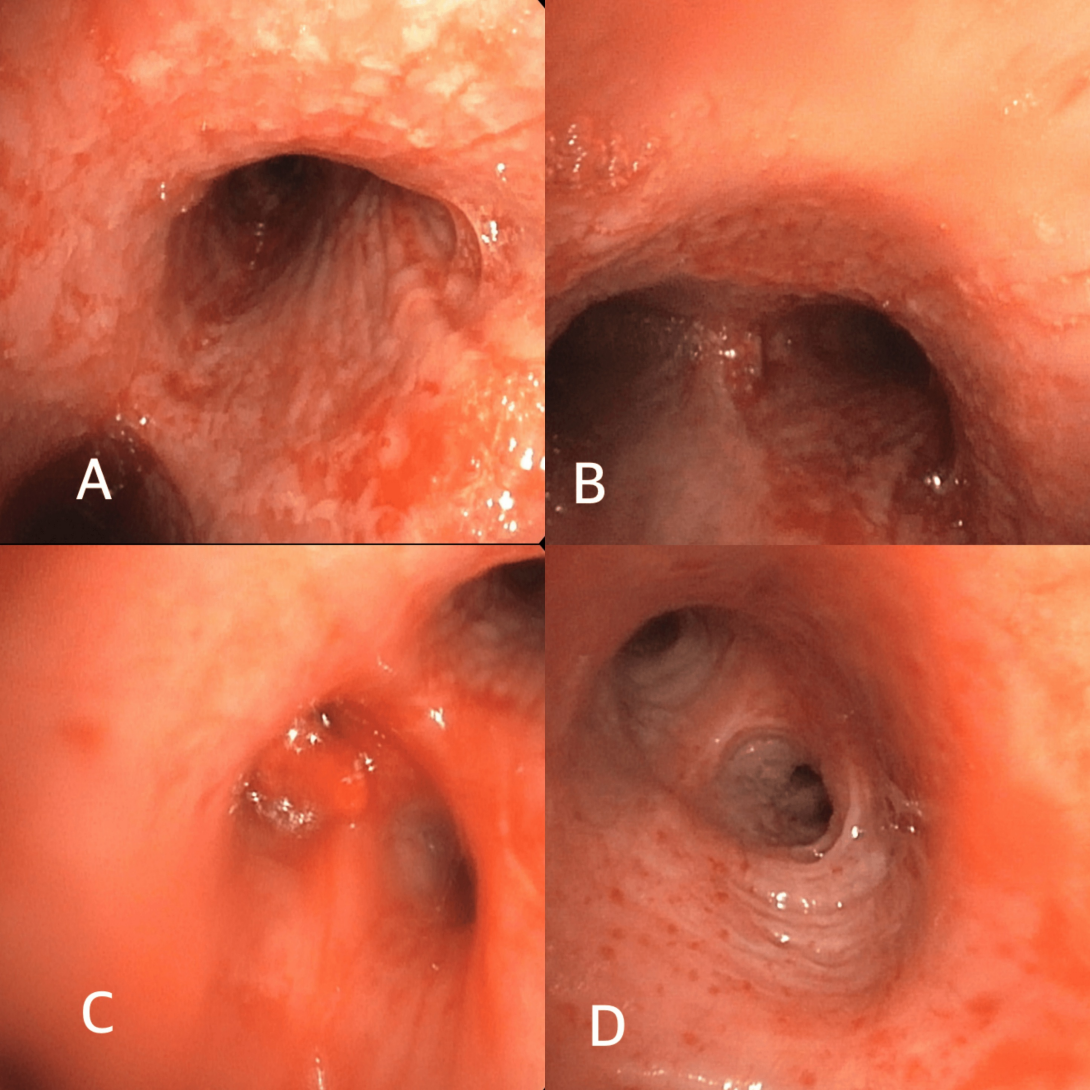
Images taken during bronchoscopy showing abnormal bronchial mucosa A) Right main bronchus; B) Carina; C) Right upper lobe; D) Left mainstem bronchus. A-D show diffuse erythematous and edematous bronchial mucosa.

As shown in Figure 2, bronchoscopic evaluation revealed friable, edematous mucosa in the left lung and cobblestoned, erythematous, friable mucosa in the right lung. Bronchoalveolar lavage cultures showed no microbial growth. Cytology and biopsy demonstrated acute inflammation without evidence of malignancy. 

An immunologic panel revealed a positive antinuclear antibody (ANA) (speckled, 1:160), positive proteinase 3 (PR3)-ANCA, and elevated cytoplasmic (C)-ANCA titers (1:160), while perinuclear (P)-ANCA, rheumatoid factor, Sjögren's syndrome type A (SSA), and Sjögren's syndrome type B (SSB) antibodies were negative. These findings, in combination with her respiratory symptoms and hemoptysis, were consistent with GPA presenting with diffuse alveolar hemorrhage. There was no clinical or laboratory evidence of renal involvement.

In Table 2, the results of the immunologic testing were compared with the results obtained in 2021 for comparison. 

**Table 2:** Results of immunologic panel ANA: antinuclear antibodies; anti-PR3: anti-proteinase 3 antibodies; C-ANCA: cytoplasmic antineutrophil cytoplasmic antibodies; P-ANCA: perinuclear antineutrophil cytoplasmic antibodies; RF: rheumatoid factor; Atypical P-ANCA: atypical perinuclear antineutrophil cytoplasmic antibodies; CRP: C-reactive protein; Anti-SSA/SSB: Sjögren's syndrome type A and B antibodies

Test	Reference range and unit	2021	2025
ANA	Negative (<1:80 titer)	Homogeneous (1:320 titer)	Speckled (1:160 titer)
PR3	0.0-0.9 units	-	7.8 units
C-ANCA	Negative (<1:20 titer)	-	(1:160 titer)
P-ANCA	Negative (<1:20 titer)	-	<1:20 titer
RF	0-14 IU/mL	<10 IU/mL	14 IU/mL
Atypical P-ANCA	Negative (<1:20 titer)	-	<1:20 titer
CRP	0.0-0.9 mg/dL	0.8 mg/dL	19 mg/dL
Anti-SSA/SSB	0.0-0.9 AI	-	<0.02 AI

A CT-guided fine-needle aspiration of the right upper lobe lesion was performed, and pathology confirmed necrotizing granulomatous inflammation.

Induction therapy was started, and the patient was treated with pulse-dose intravenous corticosteroids followed by rituximab.

**Figure 3:**
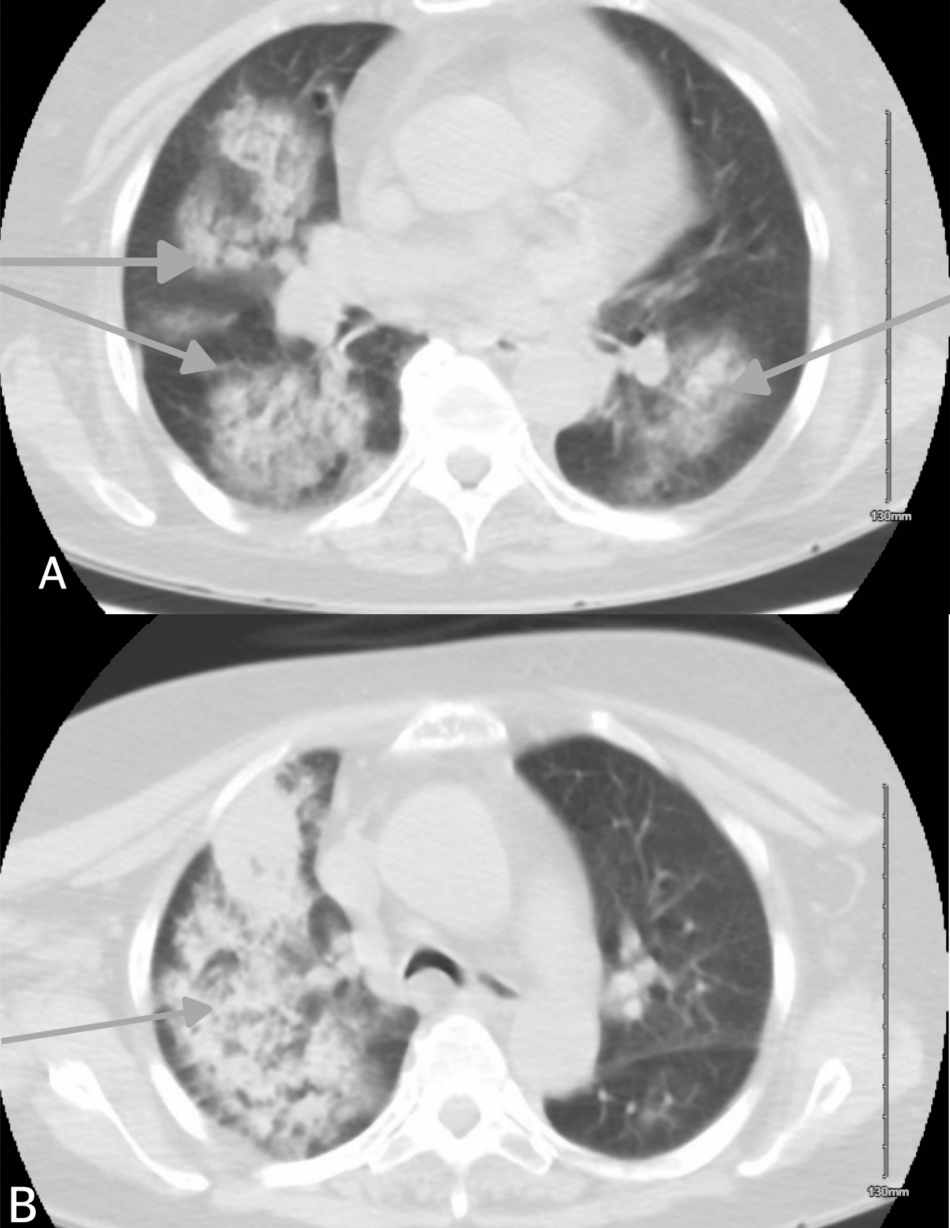
Axial view, lung window, of repeat computed tomography scan showing progression of multilobar infiltrates (gray arrows) A) Axial CT of the chest at the lower lobes/bases, showing bilateral patchy airspace opacities with a mix of ground-glass and consolidation changes. The opacities are multifocal and peripheral. B) Axial CT of the chest showing predominantly right upper lobe confluent patchy consolidation with surrounding ground-glass opacities, with the left upper lobe relatively well aerated. CT: computed tomography

Seven days after presentation, repeat imaging demonstrated near-resolution of the initial mass-like consolidation, now replaced by diffuse bilateral multilobar opacities, as seen in Figure 3. In spite of these findings on CT, the patient demonstrated significant symptomatic and clinical improvement, with resolution of shortness of breath and hemoptysis, and overall improvement in fatigue and energy level. 

Steroid taper was initiated. The patient was transitioned from high-dose intravenous methylprednisolone to 80 mg of intravenous methylprednisolone three times daily for three days, followed by oral prednisone at 1 mg/kg/day at discharge as part of the induction therapy.

At outpatient follow-up two weeks later, the patient demonstrated continued clinical improvement with complete resolution of hemoptysis and subsequently received a second dose of rituximab 15 days after the first dose. She was also initiated on mycophenolate mofetil as maintenance therapy to continue the gradual taper of corticosteroids.

At five months of follow-up, her C-ANCA titers had decreased to <1:20, and anti-PR3 antibody levels were <0.5, consistent with a favorable treatment response.

## Discussion

The relationship between infection and autoimmunity has long been recognized, with viral illnesses acting as potential triggers for the transition from subclinical immune dysregulation to overt autoimmune disease. In the context of COVID-19, this phenomenon has gained renewed attention. The present case is particularly notable in that the patient had features suggestive of underlying, yet previously uncharacterized, autoimmune activity, which rapidly evolved into clinically overt GPA following SARS-CoV-2 infection.

This progression supports the concept that COVID-19 may act not only as a trigger of de novo autoimmunity but also as an amplifier of pre-existing immune disorders. Patients with subclinical autoimmune tendencies - manifesting as nonspecific symptoms, intermittent inflammatory markers, or undifferentiated clinical findings - may remain stable for prolonged periods until an environmental trigger precipitates full disease expression. SARS-CoV-2 infection, through its profound effects on the immune system, may serve as such a trigger by tipping the balance from immune tolerance to pathogenic autoimmunity.

SARS-CoV-2 infection is associated with heightened cytokine production, disrupted innate and adaptive immune responses, and the generation of autoantibodies [1,2]. Proposed mechanisms include molecular mimicry, bystander activation, and epitope spreading, all of which may promote autoreactive lymphocyte activation in predisposed individuals [5,6], potentially accelerating the development of clinically apparent autoimmune disease.

GPA, an AAV characterized by necrotizing granulomatous inflammation and small-vessel involvement, has well-established links to environmental and infectious triggers [3,4]. The development of ANCAs, particularly PR3-ANCA, is thought to play a central role in disease pathogenesis through neutrophil activation and endothelial injury. Emerging reports have described cases of AAV following COVID-19 infection, many presenting with severe manifestations such as pulmonary-renal syndrome and rapidly progressive glomerulonephritis [7,8]. These observations further support the hypothesis that SARS-CoV-2 may precipitate vasculitic disease, particularly in susceptible hosts.

## Conclusions

This case underscores the growing, but still not fully understood, association between COVID-19 and the development or unmasking of GPA. While an increasing number of reports describe temporal relationships between SARS-CoV-2 infection and AAV, a direct causal link has not yet been established. Current evidence remains largely observational, and the mechanisms underlying this association are still being elucidated. In this case, the patient’s prior history of unexplained, chronic, and multisystem symptoms raises the possibility of a pre-existing, smoldering autoimmune process that had not yet fulfilled diagnostic criteria for a defined condition. The temporal proximity to COVID-19 infection, followed by a rapid progression to overt GPA, suggests that SARS-CoV-2 may have acted as a “second hit,” accelerating disease evolution and unmasking a previously subclinical disorder. This observation is consistent with the multi-hit hypothesis of autoimmunity, in which genetic susceptibility and baseline immune dysregulation require an external environmental trigger to precipitate clinically apparent disease. Potential mechanisms linking COVID-19 to autoimmune activation include immune dysregulation, molecular mimicry, bystander activation, and persistent inflammatory signaling; however, these remain theoretical and require further validation. Importantly, distinguishing coincidence from causation remains challenging, particularly given the background incidence of autoimmune diseases and the widespread global exposure to SARS-CoV-2.

Overall, this case adds to the accumulating body of literature suggesting an association between COVID-19 and both the induction and unmasking of autoimmune conditions, including GPA. It highlights the importance of maintaining clinical vigilance for evolving autoimmune phenomena in patients with recent SARS-CoV-2 infection, especially those with prior unexplained systemic symptoms. Further research is needed to clarify pathogenic mechanisms, establish causality, and identify individuals at increased risk for post-infectious autoimmune complications.
